# Potential of PKM2 as a drug target in mouse models with type 1 diabetes mellitus

**DOI:** 10.1002/iid3.593

**Published:** 2022-03-14

**Authors:** Junbin Liu, Zhixia Li, Gan Huang, Zhiguang Zhou, Peilin Zheng

**Affiliations:** ^1^ Department of Metabolism and Endocrinology The Second Xiangya Hospital of Central South University Changsha Hunan China; ^2^ Key Laboratory of Diabetes Immunology, Central South University, Ministry of Education National Clinical Research Center for Metabolic Diseases Changsha Hunan China; ^3^ Department of Endocrinology, Shenzhen People's Hospital, The First Affiliated Hospital of Southern University of Science and Technology The Second Clinical Medical College of Jinan University Shenzhen Guangdong China

**Keywords:** inflammation, oxidative stress, PI3K/AKT, PKM2, type 1 diabetes mellitus

## Abstract

**Background:**

This study aimed to determine the effect of PKM2 knockout in STZ induced type 1 diabetes mellitus (T1D) mouse models and to explore the possible mechanism.

**Method:**

PKM2fl/fl C57BL/6 mouse was backcrossed with Ins‐1cre C57BL/6 mouse to generate β‐cell‐specific PKM2 knockout mouse after tamoxifen administration. The expression level of PKM2 in pancreas tissues was detected by quantitative reverse‐transcription polymerase chain reaction and western blot analysis. The blood glucose levels in STZ induced T1D mouse models were measured to validate the establishment of T1D models. The pathological changes of T1D mouse were examined by hematoxylin and eosin. The oxidative stress (OS) and inflammatory response in T1D mouse were determined by measuring the expression levels of malondialdehyde, superoxide dismutase, and 8‐OHdG in pancreatic tissues and the serum levels of interleukin‐6 and tumor necrosis factor‐α. The ability to catabolize glucose was assessed through intraperitoneal glucose tolerance test and insulin tolerance test.

**Results:**

β‐cell‐specific PKM2 knockout was successfully achieved in PKM2fl/flcre+ mouse. T1D mouse with PKM2 knockdown had decreased blood glucose level and suppressed cell apoptosis. PKM2 knockout in T1D mouse attenuated β cell injury. OS and inflammatory response in T1D mouse with PKM2 knockout were also suppressed compared with T1D mouse without PKM2 knockout.

**Conclusion:**

PKM2 knockout in T1D mouse can attenuate OS and inflammatory response as well as decrease blood glucose level, suggesting the potential of PKM2 as a drug target for T1D treatment.

## Graphical abstract

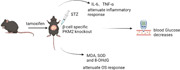

PKM2 knockout in T1D mouse can attenuate OS and inflammatory response as well as decreasing the glucose of T1D mouse

## 1 INTRODUCTION

Type 1 diabetes mellitus (T1D) is a heritable heterogeneous disease, mainly characterized by autoimmune‐mediated destruction of the pancreatic β‐cells and consequently insulin deficiency.^1^ β‐cells play an essential role in the auto‐destructive mechanisms of T1D and several interventions have been proposed to preserve β‐cells in T1D.^2^, ^3^ The diagnosis of diabetes is based on criteria of fasting blood glucose concentration above 7.0 mmol/L, a random blood glucose concentration above 11.1 mmol/L, or an abnormal result after an oral glucose tolerance test (OGTT). As a heritable polygenic disease, T1D can be affected by both environmental and genetic factors. Recent data showed this disease is more inclined to people at a young age and fortunately, the life expectancy of T1D patients has substantially increased within the past decades ascribed to the availability of exogenous insulin.^4^ Although T1D is still an incurable disease, medical development in recent years witnessed the development of gene therapy as one of the promising therapeutic alternatives for T1D.^2^, ^5^, ^6^

Pyruvate kinase (PK) is an important enzyme for glycolysis and M2 subtype PKM2 is closely associated with embryogenesis, tissue repair, and cancer initiation.^7^ The tyrosine phosphorylation of glycolytic enzymes results in increased activity of glycolytic enzymes and therefore contributes to the increased glycolytic rate and tumor cell proliferation.^8^ In this regard, it is not a surprise to notice the essential role of PKM2 in the Warburg effect.^9^ In cancer cells, PKM2 in dimer forms can enter the nuclear to regulate gene expression, thus regulating the cell biological activity and epithelial to mesenchymal transition (EMT) of cancer cells.^7^ We speculated that PKM2 may also affect the glucose metabolism in diabetes as it has been proved that in individuals with T1D history for more than 50 years, elevated glycolytic enzymes and reduced toxic glucose metabolites in renal glomeruli is connected to the preservation of kidney function.^10^ However, no study reported the direct implication of PKM2 in T1D.

Pancreatic β cell dysfunction is of paramount importance for blood glucose control and is also essential to the etiology of diabetes.^11^ Mitochondrial oxidative stress and endoplasmic reticulum (ER) stress were implicated in pancreatic β cell loss and impaired insulin secretion.^12^ Evidence from a previous study showed that in tumor cells, PKM2 regulates oxidative stress‐induced apoptosis by stabilizing Bcl‐2.^13^ In addition, PKM2 is a key factor mediating Th17 cell differentiation and autoimmune inflammation by fine‐tuning STAT3 activation.^13^ Therefore, this study aimed to explore the impact of PKM2 on β‐cells and blood glucose levels in STZ‐induced T1D mouse models.

## 2 MATERIALS AND METHODS

### 2.1 PKM2 gene knockout mouse model and T1D mouse model

Pkm2^f/f^ mice (JAX: 024048) and MIP‐Cre/ERT mice (JAX: 024709) were purchased from Jackson Laboratory and housed in cages at the temperature of 22°C–26°C, humidity of 50%–80% under a 12 h/12 h light and dark cycle. Standard diets and water are accessible for each mouse. The animal experimental procedures were approved by the Animal Research Committee of Center of Xiangya Second Hospital and were conducted based on the Guide for the Care and Use of Laboratory Animals (National Institutes of Health Publication, 8th Edition, 2011).

Pkm2^f/f^ mice (JAX: 024048) were backcrossed to a congenic C57BL/6J background for five generations, which were then used to breed with MIP‐Cre/ERT mice (JAX: 024709) to generate β‐cell‐specific Pkm2‐deleted mice (MIP‐Cre/ERT;Pkm2^f/f^, 6–8 weeks). For the induction of Pkm2 deletion, MIP‐Cre/ERT;Pkm2^f/f^ (PKM2^fl/fl^cre^+^ group), and Pkm2f/f control (PKM2^fl/fl^cre^−^ group) mice were injected with tamoxifen (20 mg/ml; T9262, Sigma‐Aldrich) for 5 days.

Seven days later, mice in PKM2^fl/fl^cre^+^‐T1D group (T1D mice with PKM2 knockout) and PKM2^fl/fl^cre^−^‐T1D group (T1D mice with Pkm2^f/f^ control) received intraperitoneal injection of 40 mg/kg STZ^14^ for 5 days consecutively to induce T1D. On parallel, mice in PKM2^fl/fl^cre^+^ group and PKM2^fl/fl^cre^−^ group were subjected to intraperitoneal injection of sterile sodium citrate‐hydrochloric acid buffer (0.05 M). The blood glucose of the caudal vein was determined by blood glucose meter (Sinocare). The establishment of T1D models was confirmed by two consecutive tests on blood glucose of 250 mg/dL (13.9 mM).^15^ The glucose in tail was measured using a glucometer after mice fasted for 6 h.

Mice in each group were euthanized with pentobarbitone. A blood sample was collected from the angular vein to collect serum and the pancreas was isolated.

### 2.2 H&E staining

The pancreas tissues were made into paraffin sections and part of the sections was dewaxed with xylene (5 μm of tissues). After that, the sections were washed in degraded ethanol (100%, 95%, 80%, and 75%) for five times and stained with hematoxylin (2 g/L) for 5 min before being washed in distilled water. Then the sections were soaked in hydrochloric acid/ethanol (1 ml: 99 ml of 70% ethanol) for 30 s and then immersed in distilled water for 15 min, followed by staining in eosin solution (1%) for 2 min and washing in distilled water. Sections were dehydrated in absolute ethanol and sealed by neutral resins. The morphology was observed under a microscope (NIKON DS‐U3, X400).

### 2.3 Immunofluorescence of pancreas tissues

For immunofluorescence, sections were deparaffinized and stained with anti‐insulin (ab181547, Abcam) and anti‐glucagon (ab75240, Abcam) antibodies followed by fluorophore‐conjugated secondary antibodies (Servicebio). The area for β cells or α cells was detected using ImageJ software (National Institutes of Health).

The apoptotic cells in pancreas tissues were detected by terminal deoxynucleotidyl transferase dUTP nick end labeling (TUNEL) kit (C1091, Beyotime). The paraffin‐embedded sections were dewaxed and washed in PBS for 5 × 3 times before 2% protease K was added for digestion for 30 min and phosphate‐buffered saline (PBS) wash for 5 × 3 times. Then the sections were incubated with mixture of TUNEL solution and insulin primary antibody (ab181547, Abcam) for 30 min at 37°C, after that the sections were washed in PBS for 5 × 3 times and then incubated with POD peroxidase‐labeled reagent for 30 min at 37°C. PBS washing was continued for 5 × 3 times and the fresh 3ʹ‐diaminobenzidine solution was prepared. The sections were observed under a microscope for 2–6 min and washed with running water before incubation with secondary antibody (ab150115, Abcam), and staining with 4′,6‐diamidino‐2‐phenylindole (D9542, Sigma). After TUNEL staining, the sections were observed under a light microscope. Five fields were selected and the apoptotic cells were counted using ImageJ software. The ratio of apoptotic cells was counted and averaged.

### 2.4 Intraperitoneal glucose tolerance test (IPGTT) and insulin tolerance test (ITT)

Intraperitoneal glucose tolerance test (IPGTT) was performed before mice were killed. After fasting for 12 h, mice in each group received intraperitoneal injection of glucose (2.0 g/kg). The blood sample from caudal vein was collected respectively at 0 min, 15, 30, 60, 90, and 120 min after the injection.

Insulin tolerance test (ITT) was performed by intraperitoneal injection of insulin (1 mU/g) after fasting for 4 h with the same way that previously described. The blood sample from the caudal vein was collected for measurement of blood glucose respectively at 0 min, 15, 30, 60, 90, and 120 min after the injection.

### 2.5 ELISA

The mice were narcotized by diethyl ether and the blood from eye socket was collected for preparation of serum. The inflammatory response was determined by measuring the serum levels of interleukin‐6 (IL‐6) and tumor necrosis factor‐α (TNF‐α) using the IL‐6 kit (PI326, Beyotime) and the TNF‐α kit (PT512). The standard sample, as well as serum and samples for positive control, was placed into the enzyme‐linked immunosorbent assay (ELISA) plates for incubation with 100 µl of antibody at room temperature at a shaking table before centrifugation at 700–900 rpm for 60 min. After incubation, the plates were washed for 4–6 times and dried. Then 100 µl substrate solution was added in each well for incubation for 15 min and the incubation was terminated by adding 100 µl of stopping buffer for reaction for 5 min. The serum levels of targets were measured on an ELISA kit.

### 2.6 Detection on index of reactive oxygen species (ROS)

Pancreatic tissue homogenate was prepared to detect the contents of malondialdehyde (MDA), 8‐OHdG, and superoxide dismutase (SOD) using MDA kit (S0131S, Beyotime), 8‐OHdG kit (ab201734, Abcam), and SOD kit (S0086, Beyotime) based on instructions of manufacturers.

### 2.7 RT‐qPCR

Pancreatic tissues were dissolved in 1 ml Trizol (Thermo Fisher Scientific) for extraction of total RNA. The total RNA was reverse transcript into complementary DNA (cDNA) with the High Capacity cDNA Reverse Transcription Kit (Thermo Fisher Scientific). Then polymerase chain reaction was performed using Real‐Time quantitative polymerase chain reaction (RT‐qPCR) and Power Sybr green PCR master mix (Thermo Fisher Scientific) on a Lightcycler 480 (Roche). GAPDH was used as the internal control. Data analysis was performed using 2^‐ΔΔCt^ method.^16^ The primer sequences were used: mouse PKM2 (Forward: 5ʹ‐CCT TCA GGA AGA CAG CCA AG‐3ʹ; Reverse: 5ʹ‐AGT GCT GCC TGG AAT CCT CT‐3ʹ).

### 2.8 Western blot analysis

The protein samples were diluted with 5× loading buffer and then subjected to electrophoretic concentration for 30 min and separation for 70 min. The reaction was terminated in PBS blocking buffer which contains 5% (w/v) skim milk powder for incubation at room temperature for 30 min. After that, the proteins were incubated overnight with primary antibody of PKM2 (1:500, ab137852, Abcam). After PBS washing, the membranes were incubated with secondary antibody for 1 h at room temperature. The blots were photographed under a Gel Doc XR Imaging System (BIO‐RAD).

### 2.9 Statistical analysis

SPSS13.0 was used for data analysis and the recorded data were expressed as mean ± standard deviation. Comparisons among groups were analyzed using a one‐way analysis of variance (ANOVA). *p* < .05 was considered as having statistical significance.

## 3 RESULTS

### 3.1 PKM2 was undetected in pancreas of PKM2 knockout mice

**Figure 1:**
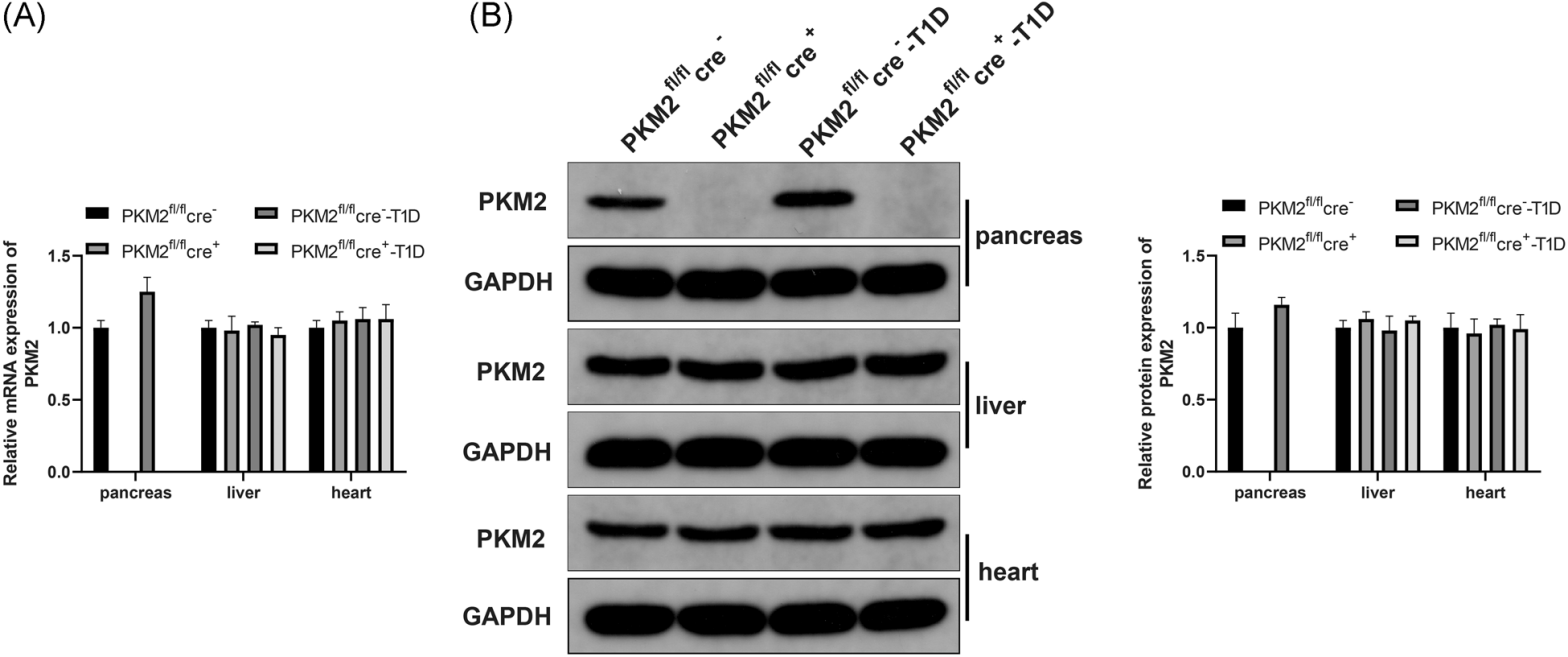
Measurement on PKM2 expression level in the pancreatic tissue and other organs of mice after PKM2 knockout. RT‐qPCR (A) was used to detect the levels of PKM2 in the pancreatic tissue, liver, and heart. PKM2^fl/fl^cre^−^: mice for control; PKM2^fl/fl^cre^+^: mice with PKM2 knockout; PKM2^fl/fl^cre^−^‐T1D: mice with T1D. T1D: Type 1 diabetes mellitus. *N* = 3. Western blot (B) was applied to detect the expression levels of PKM2 in the pancreas, liver, and heart tissues. PKM2^fl/fl^cre^−^: mice for control; PKM2^fl/fl^cre^+^: mice with PKM2 knockout; PKM2^fl/fl^cre^−^‐T1D: mice with T1D; PKM2^fl/fl^cre^+^‐T1D: T1D mice with PKM2 knockout. T1D: Type 1 diabetes mellitus. *N* = 3. RT‐qPCR, quantitative reverse‐transcription polymerase chain reaction

To verify the PKM2 knockout in mouse, we used RT‐qPCR and western blot analysis to detect the expression of PKM2 in the pancreas, liver, and heart tissues of mice. The detection showed PKM2 was not expressed in pancreas tissues of mice in PKM2^fl/fl^cre^+^ group, while mice in PKM2^fl/fl^cre^−^ group had abundantly expressed PKM2 levels in the pancreas tissues (Figure 1A,B, *p* < .05). The expression levels of PKM2 in the liver and heart tissues among the groups showed no significant difference (*p* > .05). The above results showed PKM2 knockout was successfully achieved in mouse.

### 3.2 PKM2 knockout in STZ‐injected mice maintain normal glucose levels and suppresses cell apoptosis

**Figure 2:**
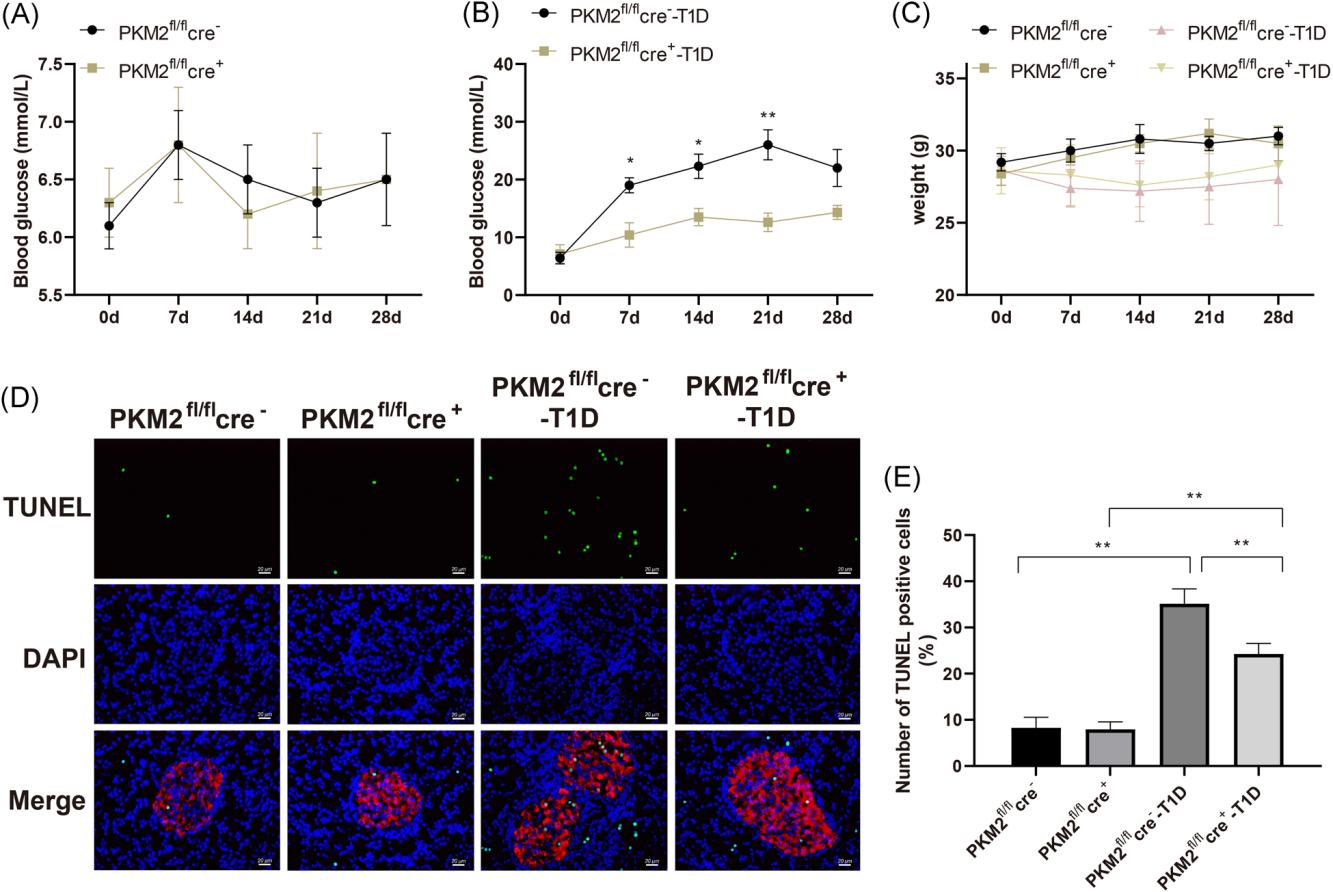
PKM2 knockout protects T1D mice from elevated blood glucose. Blood glucose were detected at the following time points: before injection of STZ (A), 0th, the 1th, the 2th, the 3th, and the 7th week after modeling (B). Body weights were also measured on the 0th, the 1th, the 2th, the 3th, and the 7th week (C). TUNEL staining to detect the apoptosis of β‐cells, in which green fluorescence represented for apoptotic cells and red fluorescence for β‐cells, while blue fluorescence for cell nucleus (D). PKM2^fl/fl^cre^−^: mice for control; PKM2^fl/fl^cre^+^: mice with PKM2 knockout; PKM2^fl/fl^cre^−^‐T1D: mice with T1D; PKM2^fl/fl^cre^+^‐T1D: T1D mice with PKM2 knockout. T1D: Type 1 diabetes mellitus, *N* = 6. *p < .05; **p < .01. TUNEL, terminal deoxynucleotidyl transferase dUTP nick end labeling

PKM2 knockout was induced by tamoxifen injection. The comparison on blood glucose between the PKM2^fl/fl^ cre^+^ group and the PKM2^fl/fl^cre^−^ group showed no significant difference (Figure 2A, *p* > .05) and both group had normal blood glucose levels. T1D mouse models were induced by STZ, after which the blood glucose levels was monitored for consecutively 14 days to verify the model establishment. On the sixth day after STZ injection, the blood glucose of mice in the PKM2^fl/fl^cre^−^ T1D group increased significantly to the diagnostic criteria for diabetes, while the blood glucose level in the PKM2^fl/fl^cre^+^ T1D group was still within the normal range and was statistically different from that in the control group (Figure 2B, *p* < .05). On the 14th day after STZ injection, the blood glucose of mice in the PKM2^fl/fl^cre^+^ T1D group was slightly above the normal standard, which was significantly lower than that in the PKM2^fl/fl^cre^−^ T1D group (*p* < .01). This indicates that PKM2 may play a role in regulating blood glucose levels in diabetes, while knocking out PKM2 alone did not directly cause the increase in blood glucose (Figure 2B, *p* < .05). At the same time, the weight of diabetic mice in PKM2^fl/fl^cre^+^ T1D group and PKM2^fl/fl^cre^−^ T1D group showed no difference (Figure 2C, *p* > .05). The changes on weight were not correlated with the increase of blood glucose (Figure 2C, *p* > .05). TUNEL staining was applied to detect cell apoptosis with green fluorescence representing for apoptotic cells and red fluorescence for β‐cells. The comparison on cell apoptosis between PKM2^fl/fl^cre^+^ group and PKM2^fl/fl^cre^−^ group showed no significant difference, while compared with PKM2^fl/fl^cre^−^‐T1D group, the cell apoptosis in PKM2^fl/fl^cre^+^‐T1D group was attenuated (Figure 2D, *p* < .01).

### 3.3 PKM2 knockout in T1D mice attenuates β cell injury

**Figure 3:**
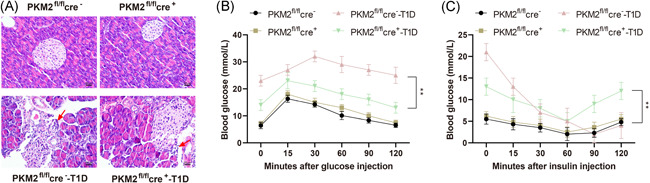
PKM2 knockout reduces the β‐cell injury in T1D mice. The injury of islet cells was assessed after H&E staining (A). Scale bar: 50 μm. The glucose tolerance was measured using intraperitoneal glucose tolerance test after fasting for 12 h (B) and the insulin tolerance test was measured by intraperitoneal injection after fasting for 4 h (C). PKM2^fl/fl^cre^−^: mice for control; PKM2^fl/fl^cre^+^: mice with PKM2 knockout; PKM2^fl/fl^cre^−^‐T1D: mice with T1D; PKM2^fl/fl^cre^+^‐T1D: T1D mice with PKM2 knockout. T1D: Type 1 diabetes mellitus, *N* = 3. *p < .05; **p < .01. H&E, hematoxylin and eosin

Injury of β‐cells is the predominant feature of T1D. To ascertain the effect of PKM2 knockout on T1D progression, we observed the morphology changes on pancreas tissues after H&E staining. Compared with PKM2^fl/fl^cre^+^ T1D group, the pancreas tissues in PKM2^fl/fl^cre^−^ T1D group had atrophic pancreas islet, dilated lumen, and infiltrated inflammatory cells (Figure 3A).

To further clarify the function of pancreatic islets in diabetic mice, after mice received intraperitoneal injection of glucose, the blood glucose was observed for 120 min to detect the ability of glucose metabolism in pancreatic islets. The glucose level in the PKM2^fl/fl^cre^−^ T1D group reached its peak ahead of that in the PKM2^fl/fl^cre^+^ T1D group, and the glucose level of PKM2^fl/fl^cre^+^ T1D group was then decreased to basic value smoothly (Figure 3B, *p* > .05). But no statistical difference was found between the PKM2^fl/fl^cre^−^ group and PKM2^fl/fl^cre^+^ group. The ITT test showed that at the 30th min, the blood glucose of PKM2^fl/fl^cre^+^ T1D group and PKM2^fl/fl^cre^−^ T1D group showed significant difference (Figure 3C, *p* < .05). The blood glucose of the PKM2^fl/fl^cre^+^ T1D group dropped to 5 mmol/L and then slowly returned to the basic value at the 90th min, while the blood glucose of the PKM2^fl/fl^cre^−^ T1D group decreased for the first 90 min to 2 mmol/L, but failed to bounce to the normal value (subsequent glucose infusion intervention measures were taken due to hypoglycemia). That result shows that PKM2 knockout can regulate blood glucose in diabetic mice.

### 3.4 PKM2 knockout suppresses T1D induced ROS and inflammation in pancreas tissues of mice

**Figure 4:**
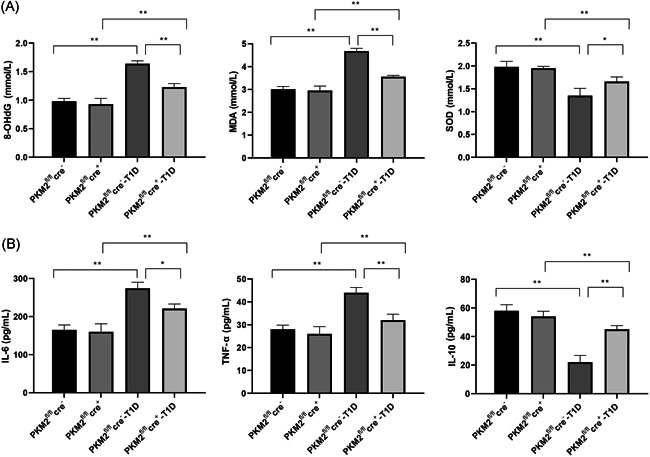
Knockout of PKM2 can suppress the release of ROS‐related factors and inflammatory cytokines. The expressions of ROS‐related factors, MDA, 8‐OHdG, and SOD in pancreas tissues (A), as well as the expressions of inflammatory cytokines IL‐6, TNF‐α, and IL‐10 in serums were measured (B). PKM2^fl/fl^cre^−^: mice for control; PKM2^fl/fl^cre^+^: mice with PKM2 knockout; PKM2^fl/fl^cre^−^‐T1D: mice with T1D; PKM2^fl/fl^cre^+^‐T1D: T1D mice with PKM2 knockout. T1D: Type 1 diabetes mellitus, ROS, reactive oxygen species. *N* = 3. *p < .05; **p < .01. IL, interleukin; MDA, malondialdehyde; SOD, superoxide dismutase

To further explore the effect of PKM2 knockout on pancreas tissues, we also measured the expression levels of reactive oxygen species (ROS)‐related proteins, MDA, 8‐OHdG, and SOD in the pancreas tissues. No significance was detected on MDA, 8‐OHdG, and SOD for the comparison between PKM2^fl/fl^cre^−^ group and PKM2^fl/fl^cre^+^ group (Figure 4A, *p* > .05). In comparison with the PKM2^fl/fl^cre^−^ group, mice in the PKM2^fl/fl^cre^−^ T1D group had increased expression levels of 8‐OHdG and MDA, and decreased expression levels of SOD (*p* < .05). Compared with mice in PKM2^fl/fl^cre^−^‐T1D group, the expression levels of 8‐OHdG and MDA were further decreased and the expression level of SOD was elevated in PKM2^fl/fl^cre^+^‐T1D group (*p* < .01). Detection on inflammatory cytokines IL‐6, TNF‐α, and IL‐10 in PKM2^fl/fl^cre^−^‐T1D group showed increased levels of IL‐6 and TNF‐α, and decreased expression level of IL‐10 when compared with PKM2^fl/fl^cre^−^ group (*p* < .01). However, no significant difference was found for comparison between PKM2^fl/fl^cre^−^ group and PKM2^fl/fl^cre^+^ group (Figure 4B, *p* > .05). Moreover, the expression levels of IL‐6 and TNF‐α in PKM2^fl/fl^cre^+^‐T1D group were decreased, while IL‐10 expression level was increased in comparison to those in PKM2^fl/fl^cre^−^‐T1D group (*p* < .01). Taken together, PKM2 knockout can attenuate T1D induced pancreatic injury by suppressing the releasing of ROS‐related proteins and inflammatory cytokines.

## 4 DISCUSSION

Better management on T1D requires a more comprehensive understanding on its pathology and etiology. Our study showed that PKM2 knockout in T1D mice can ameliorate disease progression by dampening β cell injury. In addition, the evidence in this study also supported the regulation of PKM2 on ROS and inflammatory response in T1D mice.

PKM2 is one of the rate‐limiting enzymes in glycolysis^17^ and a major player for aerobic glycolysis in cancer cells by providing energy to cancer cells.^18^ The implication of PKM2 in glycolysis is more prevalently found in cancers. For instance, in the context of breast cancer cells, PKM2 expression can be regulated through a feedback loop formed by let‐7a‐5p, Stat3, and hnRNP‐A1 to modulate glucose metabolism.^19^ Similar results can also be noticed in melanoma and the well‐known Warburg effect. In this study, PKM2 knockout was achieved in STZ induced T1D mouse models. After PKM2 knockout, we observed the morphologies of pancreas tissues in T1D mice. The observation after H&E staining showed that PKM2 knockout can alleviate the damages to islet cells. Consistently, we also found that T1D mice with PKM2 knockout have decreased glucose and increased insulin levels. Collectively, PKM2 knockout can attenuate the T1D disease progression.

In addition, further results showed that knockout of PKM2 can also decrease ROS and inflammatory response, as we found altered expression levels of inflammatory cytokines and ROS‐related factors in T1D mouse with PKM2 knockout. Normally, pancreatic β‐cells can secrete insulin in metabolic demand, such as glucose. However, persistent demand for insulin can consequently lead to progressive dysfunction and eventual loss of β‐cells.^20^ In the context of T1D, the biological reaction between immune cells and β‐cells would ultimately lead to an autoimmune assault against the β‐cells. Meanwhile, the phagocytes can release ROS to destruct the adjacent cells, which consequently lead to an unresolved inflammatory response.^20^ This information explained the presence of ROS and inflammatory response in T1D mouse, and in our study, we proved that knockout of PKM2 can attenuate the destruction to pancreatic β‐cells and therefore decrease the blood glucose levels.

To understand the mechanism of PKM2 in T1D progression, we should first understand how insulin levels can be regulated by ROS and inflammation. Evidence in a previous study showed that the inflammation‐dependent ROS from local tissues or immune cells can interact with insulin receptor (IR) and its downstream signaling pathways, resulting in failure to respond to insulin levels.^21^ The glucose transportation and storage induced by insulin signaling can be achieved with binding of insulin with IR, which in turn increases the phosphorylation of IRS and consequently activate PI3K.^22^ Similar results can be found in the previous study that PKM2 knockout can suppress the HIF1α, which may affect the transcription of genes related to glucose transporter GLUT1 and glycolytic enzymes LDHA and PDK1, thus affecting glucose uptake.^22^ The implication of PKM2 as an impact factor in signaling pathways demonstrated that knockout of PKM2 may suppress the progression of insulitis and apoptosis of islet cells.

In summary, our study highlighted the role of PKM2 in T1D progression. PKM2 knockout can attenuate T1D progression by suppressing ROS and inflammatory response. The evidence in this study suggests the potential of PKM2 in the T1D treatment in mouse models, but our study only validated the role of PKM2 in rodent experiments, more evidence is required to further validate these results in a cellular context.

## CONFLICTS OF INTEREST

The authors declare no conflicts of interest.

## AUTHOR CONTRIBUTIONS

Peilin Zheng and Zhiguang Zhou designed the research. Junbin Liu, Zhixia Li, and Gan Huang performed the research. Junbin Liu analyzed the data and wrote the manuscript.

## Data Availability

The data sets used or analyzed during the current study are available from the corresponding author on reasonable request.
